# Outer Membrane Vesicles Derived from *Escherichia coli* Induce Systemic Inflammatory Response Syndrome

**DOI:** 10.1371/journal.pone.0011334

**Published:** 2010-06-28

**Authors:** Kyong-Su Park, Kyoung-Ho Choi, You-Sun Kim, Bok Sil Hong, Oh Youn Kim, Ji Hyun Kim, Chang Min Yoon, Gou-Young Koh, Yoon-Keun Kim, Yong Song Gho

**Affiliations:** 1 Division of Molecular and Life Sciences, Department of Life Science, Pohang University of Science and Technology, Pohang, Republic of Korea; 2 Department of Emergency Medicine, College of Medicine, The Catholic University of Korea, Seoul, Republic of Korea; 3 Department of Biological Sciences, Korea Advanced Institute of Science and Technology, Daejeon, Republic of Korea; Institut de Pharmacologie et de Biologie Structurale, France

## Abstract

Sepsis, characterized by a systemic inflammatory state that is usually related to Gram-negative bacterial infection, is a leading cause of death worldwide. Although the annual incidence of sepsis is still rising, the exact cause of Gram-negative bacteria-associated sepsis is not clear. Outer membrane vesicles (OMVs), constitutively secreted from Gram-negative bacteria, are nano-sized spherical bilayered proteolipids. Using a mouse model, we showed that intraperitoneal injection of OMVs derived from intestinal *Escherichia coli* induced lethality. Furthermore, OMVs induced host responses which resemble a clinically relevant condition like sepsis that was characterized by piloerection, eye exudates, hypothermia, tachypnea, leukopenia, disseminated intravascular coagulation, dysfunction of the lungs, hypotension, and systemic induction of tumor necrosis factor-α and interleukin-6. Our study revealed a previously unidentified causative microbial signal in the pathogenesis of sepsis, suggesting OMVs as a new therapeutic target to prevent and/or treat severe sepsis caused by Gram-negative bacterial infection.

## Introduction

Sepsis is the principal cause of death in hospital populations, and its incidence has increased over the past 20 years [1], [2], [3]. The syndrome of sepsis develops when the host immune response to infection becomes excessive, which results in systemic inflammation and multiple organ failure. The inflammatory system becomes hyperactive, which involves infiltration of inflammatory cells and increased production of proinflammatory mediators such as tumor necrosis factor (TNF)-α and interleukin (IL)-6 [4], [5], [6]. Moreover, the coagulation system is triggered through extreme activation of platelets, which provokes disseminated intravascular coagulopathy [3].

Gram-negative enteric bacilli such as *Escherichia coli (E. coli)*, which are components of the normal human colonic flora, provoke sepsis through robust activation of the host immune system [7], [8], [9]. Microbial components such as lipopolysaccharide (LPS) or outer membrane proteins derived from Gram-negative bacteria can hyperactivate the host immune response via binding to pattern-recognition receptors [10], [11]. Although these soluble factors secreted from bacteria are believed to play a central role in the pathogenesis of sepsis, the exact cause of sepsis by Gram-negative bacteria is not clear.

Membrane vesicles represent nanovesicles that are secreted from cells as a mechanism for cell-free intercellular communication, which has been observed from archaea, Gram-negative bacteria, and Gram-positive bacteria to eukaryotic cells [12], [13], [14], [15], [16], [17], [18]. A wide variety of Gram-negative bacteria, including *E. coli*, constitutively secrete outer membrane vesicles (OMVs), which are defined as spherical, bilayered proteolipids with an average diameter of 20–200 nm [12], [13], [14], [15]. Previous biochemical and proteomic studies have revealed that bacterial OMVs are composed of outer membrane proteins, LPS, outer membrane lipids, periplasmic proteins, DNA, RNA, and other factors associated with virulence [14], [19], [20], [21]. Growing evidence suggests that OMVs released by Gram-negative bacteria play diverse roles in polyspecies communities by enhancing bacterial survival, killing competing bacteria, transferring genetic materials and proteins between bacterial cells, delivering toxins into host cells, and modulating the immune response in host environments.

Gram-negative bacteria involved in sepsis, such as *E. coli*, *Salmonella* and *Pseudomonas* secrete OMVs [7], [14], [22]. OMVs are enriched with LPS and outer membrane proteins known as potent immunostimulators [15], [23], and have been considered as vaccine candidates for preventing infection by Gram-negative bacteria [24], [25]. Furthermore, previous study has revealed that meningococci release many OMVs in the plasma of patients suffering from severe sepsis [26], but the physiological role of OMVs and their possible contribution to sepsis have not been defined clearly. In this study, we showed that OMVs derived from intestinal *E. coli* are causative microbial signals in the pathogenesis of systemic inflammatory response syndrome (SIRS) and sepsis-induced lethality, through the systemic induction of TNF-α and IL-6.

## Results and Discussion

**Figure 1:**
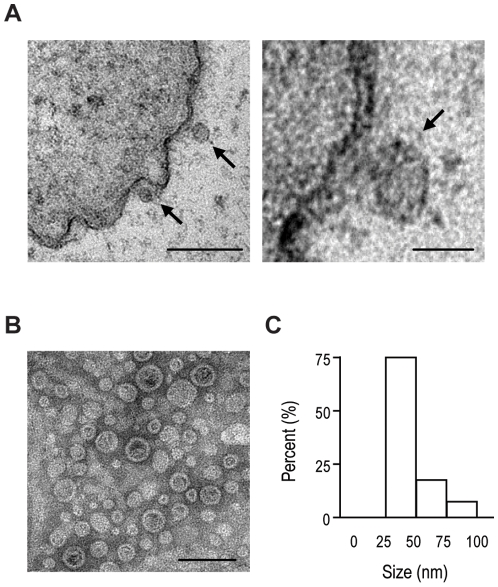
Characterization of intestinal *E. coli*-derived OMVs. **A.** Electron micrographs of thin sections of *E. coli*, showing the formation of OMVs (arrows) on the cell surface. Scale bars, 100 nm (left) and 25 nm (right). **B.** Transmission electron microscopy image of purified OMVs. Scale bar, 100 nm. **C.** Size distribution of OMVs according to diameter as determined by dynamic light scattering.

CLP (cecal ligation and puncture) is considered the most appropriate model of sepsis because it mimics the features of clinical peritonitis, including polymicrobial infection [27]. One notable feature of the CLP model is the presence of *E. coli* in the peritoneal fluid [28]. Initially, we confirmed that *E. coli* extracted from the peritoneal fluid in C57BL/6 mice after CLP, identified as *E. coli* through 16S rRNA sequencing, elaborated OMVs on their surface (**Fig. 1A**). The OMVs purified from the supernate of cultured *E. coli* were characterized by their spherical bilayered shape (**Fig. 1B**). In analysis by dynamic light scattering, the majority of the purified vesicles (75.0%) had a diameter of 25–50 nm (**Fig. 1C**).

**Figure 2:**
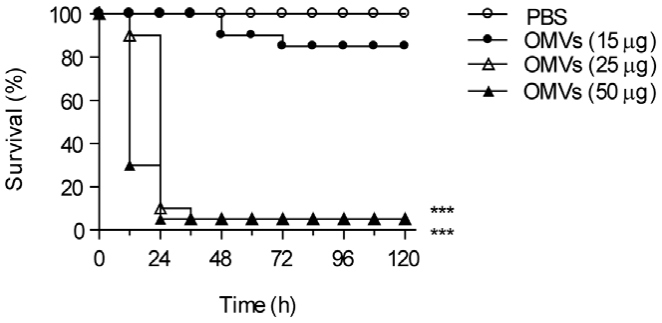
Induction of lethality in mice by intestinal *E. coli*-derived OMVs. Mice were administered intraperitoneally once with 200 µl PBS that contained 0, 15, 25, or 50 µg OMVs derived from *E. coli*. Survival was monitored every 12 h for 5 days (*n* = 20; ***P<0.001, compared to the PBS group).

We next investigated whether OMVs derived from *E. coli*, which is a commensal Gram-negative bacterium that lives in the gut, caused death in mice. OMVs were lethal when injected intraperitoneally into mice; 95% of the mice injected with 25 and 50 µg of OMVs died within 36 h after the injection (**Fig. 2**). The lethality was not a consequence of bacterial contamination because the OMV preparation was found to be sterile *in vitro*, and the peritoneum and serum of mice injected with OMVs did not contain any live bacteria (data not shown). These findings clearly indicate that OMVs derived from Gram-negative bacteria were lethal in mice.

**Figure 3:**
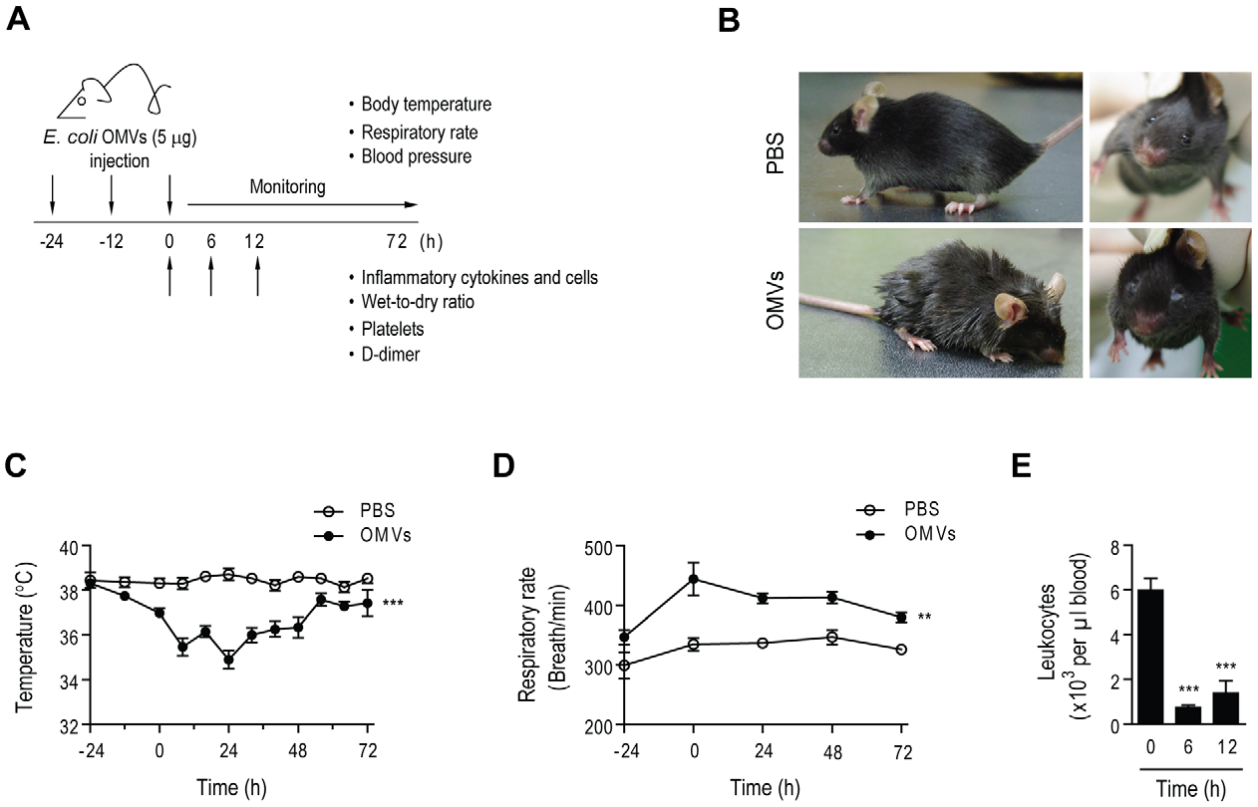
Induction of SIRS signs in mice by intestinal *E. coli*-derived OMVs. **A.** Study protocol for investigation of SIRS. Sublethal dose of OMVs (5 µg) derived from *E. coli* was injected intraperitoneally three times at 12-h intervals, and the mice were checked for SIRS index. All experiments in (**B–E**) were performed following this scheme using 5 µg OMVs derived from *E. coli*. **B.** Piloerection (left) and eye exudates (right). **C and D.** Body temperature (**C**) and respiratory rate (**D**) examined after OMVs injection (*n* = 5; **P<0.01 and ***P<0.001, compared to the PBS group). **E.** Leukocyte number in blood collected from mice after OMV injection (*n* = 5; ***P<0.001, compared to the 0 h group).

To investigate whether the OMV-induced lethality in mice was related causally to SIRS, sublethal doses of OMVs (1, 2, and 5 µg) were injected intraperitoneally three times, as shown in **Fig. 3A**, to mimic the pathophysiological conditions under which OMVs are secreted continuously. Although none of the doses induced lethality, multiple injections of OMVs (5 µg) caused eye exudates and piloerection (**Fig. 3B**). These symptoms were absent in groups treated with 1 µg of OMVs and the group injected with 2 µg only showed eye exudates (data not shown). Hence, we chose 5 µg of OMVs for the rest of the experiments.

We found a decrease in body temperature (hypothermia) after multiple injections of 5 µg of OMVs (**Fig. 3C**). Moreover, we also observed an increase in respiratory rate (tachypnea) (**Fig. 3D**) and a decrease in the number of leukocytes in peripheral blood (leukopenia) (**Fig. 3E**). Sepsis is considered positive if two or more of the SIRS criteria are met (e.g., hypothermia, tachypnea, or leucopenia [1]); therefore, our observation clearly indicates that OMVs derived from intestinal *E. coli* induced host responses which resemble a clinically relevant condition like sepsis.

**Figure 4:**
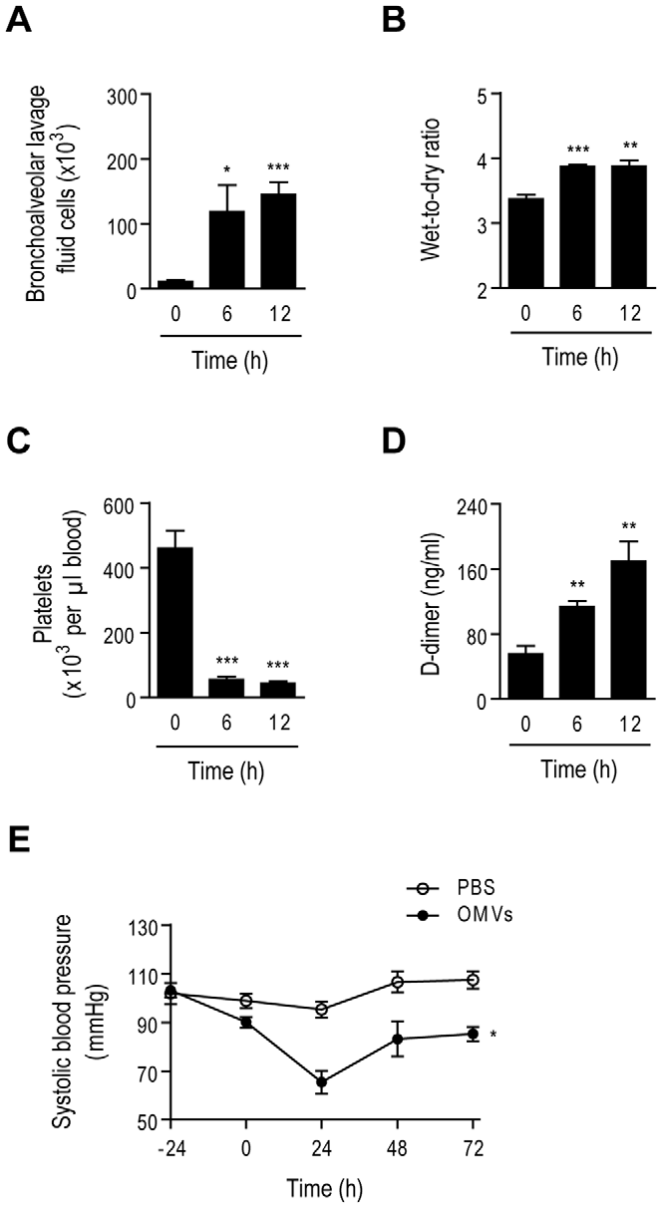
Induction of lung dysfunction, disseminated intravascular coagulation and hypotension in mice by intestinal *E. coli*-derived OMVs. **A and B.** Bronchoalveolar lavage fluid and lung organs were prepared from mice injected with OMVs derived from *E. coli* following Fig. 3A. The total number of leukocytes in bronchoalveolar lavage fluid (**A**) and wet-to-dry ratio of the lungs (**B**) (*n* = 5; *P<0.05, **P<0.01, and ***P<0.001, compared to the 0 h group). **C and D.** Measurement of the number of platelets in peripheral blood (**C**) and D-dimer levels in plasma (**D**) after OMV injection, as indicated in Fig. 3A (*n* = 5; **P<0.01 and ***P<0.001, compared to the 0 h group). **E.** Systolic blood pressure examined after OMV injection (*n* = 5; *P<0.05, compared to the PBS group).

Severe sepsis occurs when a condition induced by sepsis is associated with dysfunction of organs distant from the site of infection [29], [30]. In our study, we found that the intraperitoneally injected OMVs (5 µg) upregulated the number of infiltrated leukocytes in bronchoalveolar lavage fluid (**Fig. 4A**) and increased lung tissue permeability (**Fig. 4B**). Other notable features of severe sepsis have been linked to disseminated intravascular coagulation or hypotension [31], [32], [33]. Platelets in peripheral blood decreased (**Fig. 4C**) and D-dimer levels in plasma increased (**Fig. 4D**) after OMV treatment, which indicated that OMVs caused disseminated intravascular coagulation. Moreover, OMV injection caused a drop in blood pressure from 103.2±6.7 to 65.4±10.5 mmHg (**Fig. 4E**), which is the major determinant of lethality [31], [32].

**Figure 5:**
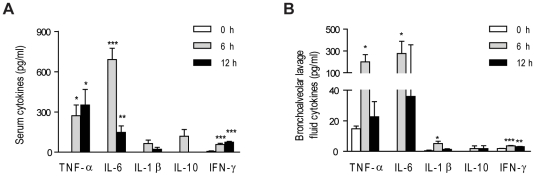
Onset of systemic inflammation by intestinal *E. coli*-derived OMVs. Measurement of cytokines levels in serum (**A**) and bronchoalveolar lavage fluid (**B**) by ELISA after OMV injection, as indicated in **Fig. 3A** (*n* = 5; *P<0.05, **P<0.01, and ***P<0.001, compared to the 0 h group).

Severe sepsis is accompanied by systemic inflammation that results from excessive release of cytokines into the systemic circulation [34], [35]. Serum levels of TNF-α and IL-6 were markedly enhanced 6 h after multiple injections of 5 µg OMVs (**Fig. 5A**). Increased levels of such cytokines were observed similarly in bronchoalveolar lavage fluid, which indicated the presence of SIRS in lung tissues (**Fig. 5B**). On the other hand, other SIRS-associated mediators, such as IL-1β, IL-10, and interferon (IFN)-γ in serum and bronchoalveolar lavage fluid showed only a slight increase compared with TNF-α and IL-6 levels. TNF-α and IL-6 are well-known to be important cytokines in the pathogenesis of sepsis, and the level of these cytokines in serum is a crucial indicator of sepsis [4], [5], [6], [36].

**Figure 6:**
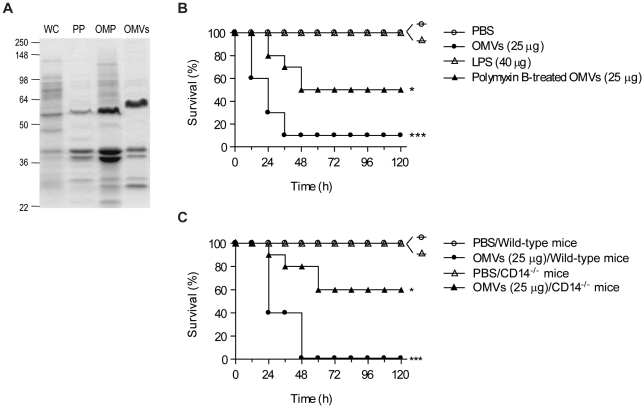
Both vesicular LPS and other vesicular components are key factors in OMV-induced lethality. **A.** Coomassie-brilliant-blue-staining of whole-cell lysates (WC), periplasmic proteins (PP), outer membrane proteins (OMP), and OMVs, each 10 µg. Molecular weight standards are indicated on the left (kDa). Note that OMVs harbor 75 ng of LPS per 100 ng of OMV proteins. **B.** Survival curve of wild-type mice from a single intraperitoneal injection of various samples as indicated (*n* = 10; *P<0.05 and ***P<0.001, compared with PBS group). **C**. Survival curve of wild-type and CD14^−/−^ mice after single injection of 25 µg OMVs (*n* = 10; *P<0.05 and ***P<0.001, compared to the PBS group).

OMVs are enriched with LPS and outer membrane proteins known as potent immunostimulators [14], [15], [19]. As reported, OMVs derived from *E. coli* were enriched with LPS and many proteins including outer membrane proteins (**Fig. 6A**); OMVs harbor 75 ng of LPS per 100 ng of OMV proteins. We further investigated the role of vesicle-associated LPS on the development of OMV-induced lethality. When OMVs (25 µg) or LPS (40 µg, about twice the amount of LPS than that in 25 µg OMVs) were injected intraperitoneally once into mice, LPS alone showed no lethality in contrast to OMVs injection (**Fig. 6B**). We also observed that co-treatment of polymyxin B with OMVs (25 µg) resulted in a decrease in OMV-induced lethality. Furthermore, the intraperitoneally injected OMVs (25 µg) caused delayed and reduced lethality in CD14^−/−^ mice than in wild-type mice (**Fig. 6C**). CD14 is the co-receptor of LPS [37]. Although further studies should be addressed, our findings support the conclusion that OMVs act as more powerful SIRS inducers than LPS alone, and that both vesicular LPS and other vesicular components including proteins should be crucial in the pathogenesis of sepsis-induced lethality.

The present study showed that intraperitoneal injection of OMVs derived from intestinal *E. coli* induced host responses which resemble a clinically relevant condition like SIRS that was characterized by piloerection, eye exudates, hypothermia, tachypnea, leukopenia, disseminated intravascular coagulation, dysfunction of the lungs, hypotension, systemic induction of TNF-α and IL-6, and lethality. Earlier *in vitro* studies showed that OMVs derived from *Pseudomomas* delivered multiple secreted virulence factors into the cytoplasm of airway epithelial cells through a lipid raft-mediated pathway [38]. Based on these observations, Bomberger *et al.* proposed the possibility that direct delivery of bacterial proteins by OMVs could occur without bacteria, even at a far distance. We observed that intraperitoneal injection of OMVs induced lung dysfunction through systemic inflammation. Thus, OMVs might be efficient enough to induce systemic inflammation in distant organs, although bacteria themselves are important for the development of sepsis at the first infection site.

Understanding the microbial factors in the pathogenesis of severe sepsis and sepsis-induced lethality is essential in developing rational strategies for prevention and treatment of sepsis caused by bacterial infection. Although LPS/endotoxin is considered the critical microbial signal in Gram-negative bacterial sepsis [10], increasing evidence suggests that outer membrane proteins are also key components in sepsis induction. Lipoproteins, which are the most abundant outer membrane proteins, can cause lethal shock in mice [11], and when combined with LPS, they can cause sepsis synergistically [39]. In addition, previous evidence indicates that OMVs derived from *Neisseria meningitidis* are more potent inducers of inflammation than purified LPS [23]. Note that OMVs are especially enriched with outer membrane proteins, including OmpA, OmpF, and NmpC, which are potent immunostimulators [19]. It is interesting to note that Gram-negative bacteria involved in sepsis, such as *E. coli*, *Salmonella* and *Pseudomonas* secrete OMVs [7], [14], [22].

In conclusion, we demonstrated that the OMVs derived from Gram-negative bacteria are previously unidentified causative microbial signals in the pathogenesis of severe sepsis and sepsis-induced lethality, through the induction of proinflammatory cytokines, particularly TNF-α and IL-6. Although further studies should be issued, our findings could lead to a better understanding of the pathogenesis of sepsis and sepsis-related diseases caused by Gram-negative bacterial infection, and could have major implications for the development of diagnostic tools, vaccines, and treatment of these syndromes.

## Materials and Methods

### Mice

We used 6–8 week old male wild-type mice and CD14^−/−^ mice of the C57BL/6 genetic background from the Jackson Laboratory. Experimental protocols were approved by the Institutional Animal Care and Use Committee at Pohang University of Science and Technology, Pohang, Republic of Korea with approval number: 2009-01-0001.

### Selection of intestinal *E. coli* after CLP

For CLP experiments, we anesthetized the mice, ligated the cecum, punctured it once with an 18-gauge needle, and closed the abdomen [40]. Forty hours after CLP, mice were anesthetized for peritoneal lavage fluid collection. The fluid was cultured overnight on Luria-Bertani agar plates at 37°C. A single colony was picked and identified through 16S rRNA sequencing using primers 27f and 1492r [41], [42].

### Preparation of OMVs from *E. coli*

Bacterial cultures grown in Luria-Bertani broth were pelleted twice at 5,000× *g* for 15 min. The supernatant fraction was filtered through a 0.45 µm vacuum filter and was concentrated by ultrafiltration with a QuixStand Benchtop System (Amersham Biosciences) having a 100-kDa hollow fiber membrane (Amersham Biosciences). Another filtration through a 0.22 µm vacuum filter was done to remove any remaining cells. OMVs were prepared by pelleting after the centrifugation in a 45 Ti rotor (Beckman Instruments) at 150,000× *g* for 3 h at 4°C. OMVs diluted in phosphate-buffered saline (PBS) were stored at −80°C [19]. The protein concentration of OMVs was assessed by the Bradford assay (Bio-Rad Laboratories). The size distribution of OMVs was measured by dynamic light scattering using Zetasizer Nano ZS (Malvern Instruments) and was analyzed by Dynamic V6 software [43].

### Transmission electron microscopy

After fixation with 2.5% glutaraldehyde, cultured bacteria were pelleted and post-fixed in 1% osmium tetroxide for 1 h, dehydrated in a graded series of ethanol, and embedded in epoxy resin. Thin sections were prepared using diamond knives (Diatome) on an MTX microtome (RMC Boeckeler Instruments), placed on 150-mesh coated copper grids (EMS) and stained with 3% uranyl acetate and lead citrate. For analysis of OMVs, OMVs in PBS were placed on 400-mesh copper grids and stained with 2% uranyl acetate. Images were obtained using a JEM1011 microscope (JEOL) at an accelerating voltage of 100 kV.

### Periplasm and outer membrane preparations

Periplasm and outer membrane were purified as described previously [44]. Briefly, spheroplasts were made from *E. coli* by lysozyme (600 µg/g cells) and 0.1 M EDTA treatment. The spheroplasts resuspended in ice-cold 10 mM Tris–HCl (pH 8.0) were sonicated and centrifuged at 8,000× *g* for 5 min. Whole membranes were pelleted from the supernates at 40,000× *g* for 1 h for outer membrane preparation. The preparation was pelleted at 40,000× *g* for 90 min after incubation in 0.5% sarkosyl (Sigma Chemical Co.) at 25°C for 20 min. Final pellets were resuspended in ice-cold 10 mM Tris–HCl (pH 8.0) and stored at −80°C. Protein samples from whole-cell lysate, periplasm, outer membrane, and OMVs were analyzed by sodium dodecyl sulfate-polyacrylamide gel electrophoresis (10% resolving gel), followed by gel staining with Coomassie brilliant blue R-250 (Sigma Chemical Co.).

### Investigation of septic signs in mice

Rectal temperature and systolic blood pressure were measured using a Digital thermometer (Natume) and a computerized tail-cuff system (Visitech Systems), respectively. Respiratory rate was measured in conscious, unrestrained mice using noninvasive whole-body plethysmography (Allmedicus). For leukocyte counting in blood, blood sample was obtained from anesthetized mice by cardiac puncture and put into EDTA-tube. Following 6 min incubation with 1% HCl, the number of cells was counted using the light microscopy.

### Evaluation of inflammatory index in mice

Serum and bronchoalveolar lavage fluid were collected from mice after treatment of OMVs. Each sample was centrifuged, and the supernates were stored at −80°C until cytokines evaluation. The pelleted cells in bronchoalveolar lavage fluid were resuspended in PBS and counted using the light microscopy. For cytokines measurements, the cytokines present in the serum and bronchoalveolar lavage fluid were analyzed by ELISA (R&D Systems).

### Wet-to-dry ratio of the lungs

After the OMVs-treated mice were killed, lungs were taken from the mice and weighed. They were weighed again after 2 days of drying at 55°C, followed by calculation of wet-to-dry ratio [45].

### Platelet and D-dimer measurements

Whole blood from heart was collected in EDTA-tube and diluted 1∶100 in 1% ammonium oxalate. Following the 10 min incubation, the number of platelet was counted using the light microscope. For D-dimer measurement, after treating the whole blood with sodium citrate, D-dimer was quantified by Asserachrom D-dimer ELISA kit (Diagnostica Stago).

### Statistical analyses

Survival curves were compared by the log-rank test. The rest of the data were analyzed by Student's *t* test using the GraphPad Prism statistical program. *P*<0.05 was considered significant. Results are expressed as means ± SEM.
